# Why has the taxing policy on sugar sweetened beverages not reduced their purchase in Iranian households?

**DOI:** 10.3389/fnut.2023.1035094

**Published:** 2023-02-06

**Authors:** Delaram Ghodsi, Arezoo Haghighian-Roudsari, MohammadReza Khoshfetrat, Seyedeh Fatemeh Abdollah-PouriHosseini, Mitra Babapour, Fatemeh Esfarjani, Marjan Ajami, Azizollaah Zargaraan, Fatemeh Mohammadi-Nasrabadi

**Affiliations:** ^1^Department of Nutrition Research, Faculty of Nutrition Sciences and Food Technology, National Nutrition and Food Technology Research Institute, Shahid Beheshti University of Medical Sciences, Tehran, Iran; ^2^Department of Community Nutrition, Faculty of Nutrition Sciences and Food Technology, National Nutrition and Food Technology Research Institute, Shahid Beheshti University of Medical Sciences, Tehran, Iran; ^3^Research Department of Food and Nutrition Policy and Planning, Faculty of Nutrition Sciences and Food Technology, National Nutrition and Food Technology Research Institute, Shahid Beheshti University of Medical Sciences, Tehran, Iran; ^4^Department of Economics, Allameh Tabataba’i University, Tehran, Iran

**Keywords:** fiscal policies, soft drinks/sugar sweetened beverages (SSBs), purchase, production, expenditure, price, tax

## Abstract

**Objective:**

This study aimed at analyzing the effectiveness of the policy of taxing Sugar-Sweetened Beverages (SSBs) on their purchases during the last decade in Iranian households.

**Methods:**

The present mixed method study was done in 2017 in four phases: (1) A meta-review of the fiscal policies during the last decade, (2) Collecting existing data on soft drinks’ production, price, and household expenditure during the last decade, (3) Conducting 19 semi-structured interviews with key informants, and (4) Facilitating a national meeting to achieve a consensus on the recommendations and future implications.

**Results:**

Document reviews showed that based on the Permanent Provisions of National Development Plans of Iran, the Ministry of Health and Medical Education (MOHME) should announce the list of health threatening products to increase taxation for them. The government is allowed to impose taxes on domestically produced and imported SSBs. The average household expenditure on SSBs increased in the rural and urban households of Iran during 2006–2016 in spite of taxation. In the different key informants’ opinion, only value-added tax (VAT) was implemented among different fiscal policies, and the other parts, including tax and tolls were debated.

**Conclusion:**

The present research findings further proposed some suggestions for increasing the effectiveness of financial policies in reducing the prevalence of NCDs in Iran.

## Introduction

Non-communicable diseases (NCDs) account for more than one-half of the global burden of diseases (1) and recent studies show that NCDs are responsible for near 70% of death worldwide (2). Furthermore, it was established that NCDs contribute to most of premature mortality in low- and middle-income countries (LMICs) (3). It is estimated that NCDs would pose accumulative global economic losses of US$ 47 trillion by 2030, approximately 75% of global gross domestic product (GDP) (3, 4). Iran has recently experienced a rapid nutrition transition (5, 6). NCDs results in many diseases in both sexes (7) and according to the World Health Organization (WHO) reports, they are responsible for 70% of all deaths in Iran (5, 8). Primary prevention is a key component of combating NCDs that can minimize exposure to risk factors, and thus, pave the way for establishing healthy behaviors (9). High blood pressure, overweight, obesity, and unhealthy diets high in sugar, salt, and fat are considered the major nutritional risk factors of NCDs that are somewhat preventable (10, 11).

Different policies are being implemented worldwide to control and prevent NCDs. The Global Action Plan for Prevention and Control of Non-communicable Diseases (2013–2020) proposes that countries consider using economic tools. Some of these policies focus on decreasing nutritional risk factors in food products. Some focus on improving the access to healthy foods choices and discouraging the consumption of less healthy foods (12, 13). There are different ways to incentivize consumers to choose healthier foods, including promotion of nutrition literacy, controlling the advertisement of food products, food labeling, and fiscal policies (14). Fiscal policies such as taxes and subsidies are increasingly considered as potential policy instruments to incentivize the consumers to improve their food and beverage consumption patterns (13, 15–21).

In order to reduce the consumption of unhealthy foods, taxation on sugar sweetened beverages (SSBs) and soft drinks have been suggested along with subsidies targeted to fruits and vegetables (22, 23). Taxation on SSBs is mentioned by the WHO’s technical report as the first effective intervention for controlling NCDs (12). Eastern Mediterranean Region (EMR) - World Health Organization (EMR-WHO) has also proposed policy priorities, including the implementation of fiscal measures to prevent NCDs and obesity in the region (24, 25). Saudi Arabia was the pioneer in implementation of tax policy (26, 27) followed by the United Arab Emirates (UAE) and Bahrain in 2017, Oman and Qatar in 2019, and Kuwait in 2020. These countries adopted a 50% tax on carbonated drinks and 100% tax on energy drinks in 2016. The introduction of health-related taxes on soft drinks has been followed by a drop in the growth rate of sales (28–30).

Fiscal policies have not been used for controlling the nutritional risk factors in Iran for a long time, whereas “nutrition transition” was intensified in the early 1980s. Food subsidies during this period increased the amount of fat and carbohydrate consumption of the households’ food baskets. Regarding the socio-economic and particular political situation of Iran in the region, the policies implemented for each food product were different during the past years. The Iranian government applied subsidies for the production and consumption of basic food products to cover the difference between high producer prices and low consumer prices (31, 32). Untargeted consumer subsidies on mainly energy providing foods to secure minimum and equitable food supplies have been reduced substantially over the last years, but remained in place for bread (33). One of the important challenges of policy makers is the dual effects of fiscal policies on food and nutrition. So, this study aims to:

(1) Reviewing the fiscal policy documents related to SSBs in Iran.
(2) Collecting existing data on SSB production, consumption, and household expenditure during the last decade in the Islamic Republic of Iran.
(3) Identifying the facilitators, barriers, and challenges of fiscal policies related to food and nutrition in Iran by conducting profound semi-structured interviews with key informants.
(4) Holding a national meeting to enhance communication between the stakeholders to advocate and achieve a consensus on the recommendations for improving the policy implementation and enhancing its impact.

## Methods

This research is a combination of review and qualitative study, which was conducted in 2017. Data were collected in four phases:

Phase 1: A meta-review of the current fiscal policies on SSBs in Iran.
Phase 2: Reviewing the secondary data on the production, consumption, and price of SSBs during the last decade.
Phase 3: Using semi-structured interview with key informants regarding the facilitators and barriers of the fiscal policies on SSBs and challenges encountered in the implementation of these policies.
Phase 4: Holding a national expert panel workshop to advocate and achieve a consensus on the recommendations and future implications.

**FIGURE 1:**
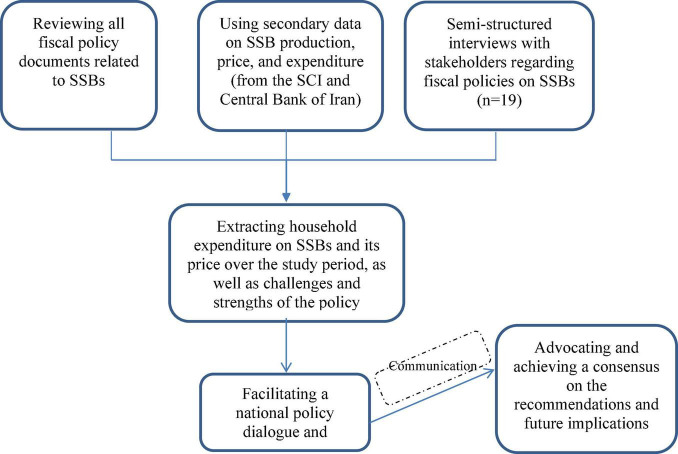
Flowchart of phases exploring the challenges and strengths of fiscal policy on SSBs in Iran and achieving a consensus on it.

Figure 1 shows the flowchart of different phases of this study.

Phase 1- A meta-review of the current fiscal policies on SSBs in Iran: All regulatory measures, documents and master plans, including the constitution, the development plans, overall health policies and approvals from the High Council of Health and Food Security, as well as subsidies and taxes regarding the trade and marketing of SSBs in Iran were collected through the websites and Google scholar search up to 2016. Reference lists of the selected articles were also manually scanned for additional eligible studies using “sugar sweetened beverages,” “tax,” “policy,” “soda,” “carbonated beverages,” and “Iran” keywords. All published and unpublished documents related to policies, regulations and programs on fiscal policies from the public and private organizations were collected and reviewed, too. The collected data were reviewed and analyzed considering their contents, weaknesses and strengths. In addition, reasons for their success, strengths, and gaps were extracted and some solutions were recommended. The progress on the proposed Sin Tax recommended by the WHO was assessed and clear recommendations highlighting the expected impacts were presented.

Phase 2- Reviewing the existing data on the production, consumption, and price of SSBs during the last decade: All data regarding the SSBs production, consumption, and household expenditure during the last decade were collected through the information gathered from the Statistical Center of Iran (SCI), Central Bank of the Islamic Republic of Iran, the Islamic Republic of Iran Customs Administration (IRICA), and Ministry of Health and Medical Education (MOHME). Tax revenue was obtained from Tax Affairs Organization affiliated with the Ministry of Economic Affairs and Finance.

The nominal price and consumer price index (CPI) of SSBs from 2011 to 2016 were obtained from the Central Bank of the Islamic Republic of Iran data based on 2011 as a basic year and those of 2017–2021 adapted from SCI based on 2016 as the basic year. To remove the price effect from data and change nominal data to real values, the deflated fixed price was estimated by dividing the nominal price data by CPI and multiplying the result by 100.

Phase 3- Using semi-structured interview with key informants regarding the facilitators and barriers of the fiscal policies on SSBs: In this phase, a qualitative study was simultaneously conducted through interviews with key informant experts about fiscal policies on reducing the consumption of SSBs, as well as identifying their weaknesses and strengths, and implementation challenges. Key informant actors (*n* = 19) were selected in each food, nutrition, and health area using purposive and snowball sampling. Sample seed diversity and persistence (within reason) were used to increase diversity in the snowball samples (34). Semi-structured in-depth interviews were applied according to the interview protocol developed by the research team based on their expertise and the literature (35–38), including open questions according to the study purpose (Appendix 1). At first, the research purpose was explained to the participants and informed consent was obtained from all of them. They were asked to introduce the other informants at the end of the interview. Each session took 1 h. The interviews were audio-recorded and transcribed verbatim; non-verbal communications and interactions were also noted. The sampling continued until the theoretical saturation was achieved.

Phase 4- Holding a national expert panel workshop to advocate and achieve a consensus on the recommendations and future implications: The key informants and policy makers, interviewed in the previous phase, were invited to participate in a 1-day expert panel workshop to be held in autumn 2017 in Tehran, Iran. They were from MOHME, National Nutrition and Food Technology Research Institute (NNFTRI), Iranian National Tax Administration (INTA), Ministry of Industry, Mine and Trade, Iran Food and Drug Administration, the WHO representative in Iran, and Institute of Standards and Industrial Research of Iran. The NNFTRI hosted the workshop, namely “Fiscal policies on healthy diet in the Islamic Republic of Iran.” The workshop’s overriding goal was to present and validate the collected data, enhance communication between the domestic policy makers, stakeholders, researchers and experts, and the WHO representative in Iran about the needs, capabilities and future directions, and also advocate and achieve a consensus on the recommendations and future implications.

### Statistical analysis

All documents related to the fiscal policies on SSBs in Iran were content analyzed to explore their weaknesses, strengths, and contents. Data related to SSBs production, consumption, tax, and price index during the last decade were presented as charts using the Microsoft Excel (ver. 2016). All interviews, discussions and debates that took place at the workshop were recorded and transcribed verbatim. All transcribed interviews were simultaneously analyzed together with data gathering using the MAXQDA 10 software. These data were then content analyzed to identify the experts’ and key informants’ opinions regarding the present situation and future directions.

## Results

### Regulations on SSBs

**FIGURE 2:**
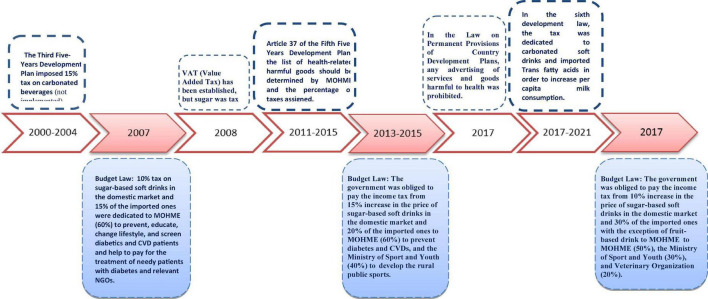
Timeline and key milestones of the formulation and adoption of the carbonated soft drinks/sugar sweetened beverages tax in Iran. MOHME, Ministry of Health and Medical Education; CVDs, cardiovascular diseases; NGOs, non-governmental organizations.

Timeline of the formulation and adoption of SSBs tax in Iran is illustrated in Figure 2. The review of all documents (*n* = 22) on the taxation of health threatening products in Iran showed that, for the first time, Iran’s Third 5-year Development Plan (2000–2004) imposed a 15% tax on SSBs; however, it was not implemented due to operational problems. VAT (Value Added Tax) policy was enacted in 2008; however, wheat flour, bread, meat, sugar, rice, legumes and soybean, milk, cheese, vegetable oil, and dry milk were tax-free. Article 37 of Iran’s Fifth 5-year Development Plan (2011–2015) was approved for preventing and controlling the diseases and health-related risk factors with the most economic and social costs. Based on this article, MOHME is responsible for determining and introducing the list of health threatening products and drugs with a potential for abuse. The percentage of charges for these commodities should be determined and notified at the beginning of each year by a working group under the responsibility of MOHME, with the membership of the Ministry of Economic Affairs and Finance, Ministry of Commerce, Welfare and Social Security, and Ministry of Industries, Mines and Trade, as well as the Vice President of Strategic Planning and Control.

**FIGURE 3:**
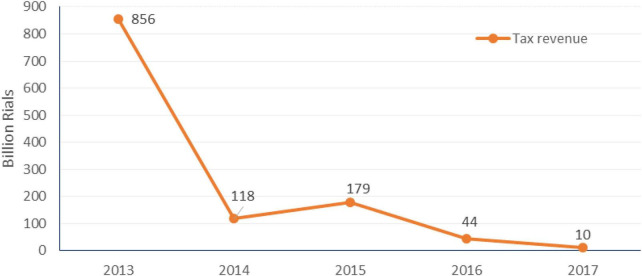
Sugary soft beverage tax revenue from 2013 to 2017 in Iran.

According to the Budget Law of 2013, which was published based on the Iran’s Fifth 5-Year Development Plan, the government is obliged and started to receive the tax revenue for 15% increase in the price of domestically produced SSBs and 20% increase in the price of imported SSBs (Figure 3). Sixty percent of the tax revenue should be paid to MOHME for prevention and treatment of diabetes and 40% to the Ministry of Sports and Youth to promote physical activity in rural areas. In the Sixth 5-Year Development Plan of the Islamic Republic of Iran, this tax was dedicated to SSBs and imported Trans fatty acids. Based on the Iran Parliament approval (June 2021), the amount of tax and duty on domestically produced and imported SSBs increased to 16 and 36%, respectively in 2021. After the first year of implementing the tax policy, SSBs industry owners filed a lawsuit in 2013, and the investigation and announcement of the verdict rejecting the lawsuit and its implementation took a long time until 2015 (39). In 2021, the Honorable Board of Ministers approved a letter of approval stating that according to the Law of the Sixth Five-Year Program of Economic, Social and Cultural Development of the Islamic Republic of Iran - approved in 2016 - the amount, the method of determining and the authority for setting taxes on carbonated drinks have changed and the budget law has been reflected, but so far the authority has not been established and taxes has not been established, therefore, it is not possible to claim taxes based on the budget law and the taxes subject to the country’s budget law of the year 2013 regarding SSBs from 2014 to 2019 will be canceled and will not be claimed (40).

Document review showed that the High Council of Health and Food Security as an authority for approving food and nutrition laws has already approved some regulations on advertising and consumption of SSBs but nothing about their taxation.

### SSB price index, tax revenue, and expenditure in Iran

**FIGURE 4:**
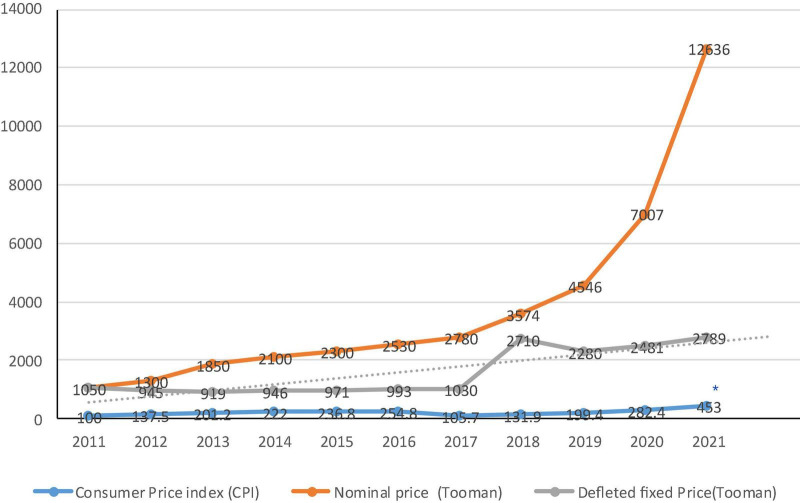
The consumer price index, nominal price, and deflated fixed price of 1.5-liter carbonated soft drinks (Tooman) in Iran during 2011 – 2021. *The consumer price index from 2011 to 2016 was obtained from the Central Bank of Iran based on 2011 as a basic year and those of 2017–2021 adapted from SCI based on 2016 as the basic year.

The highest tax revenue from SSBs tax was obtained in 2013 when the taxation on SSBs was passed. The tax revenue has decreased drastically after almost 1 year of taxation policy enacted in 2013 (Figure 3). The deflated price trend showed a gradual increase from 2011 to 2016; however, it had a sharp increase later such that the average price of 1 liter of SSBs in 2017 was US$ 0.40–0.47 in Tehran market (Figure 4).

**FIGURE 5:**
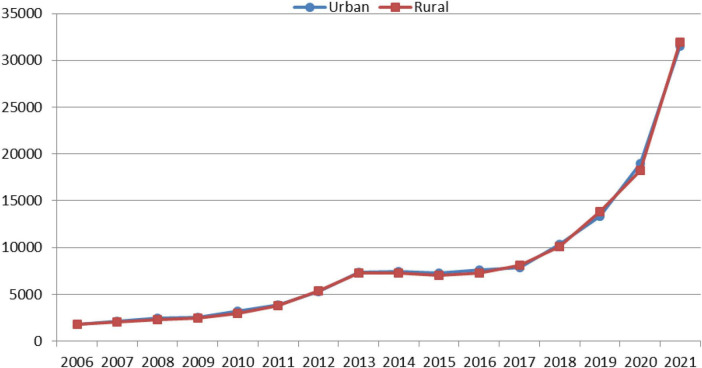
Average per capita monthly Iranian household expenses on sugary soft beverages by their residency (2006–2021).

Data obtained from MOHME showed that Iranians consumed 32–42 liters of SSBs per capita in 2016. According to the Customs authority data, 6.3 million liters of SSBs were imported to Iran in the first 10 months of 2016, mostly from Turkey. In addition, only 1% of domestically produced SSBs are exported and 99% are consumed in the country. Data analysis of the Central Bank of the Islamic Republic of Iran and ISC demonstrated that the average per capita household expenses on SSBs increased in both the rural and urban households from 2006 to 2013. It remained almost constant until 2016 and then increased again (Figure 5).

Other results also showed a lower expenditure on SSBs in the lower deciles of rural households comparing to urban households. However, these amounts were higher in the 8th income decile rural households comparing to their similar urban counterparts.

### Key informants’ opinions on fiscal policies

Overall, 19 semi-structured interviews were conducted with key informants. The general characteristics of the interviewees are shown in Table 1.

**TABLE 1:** General characteristics of the participants in the interview sessions.

No.	Position	Representative for	Organization
1	Deputy	Government	General Directory for Food and Beverage Products, Iranian Food and Drug Administration (IFDA)
2	Head	Government	The Marine and Animal Food Department, IFDA
3	Head	Government	Plant-based Food Department, IFDA
4	General Director	Government	Deputy of Industries’ Affairs, Ministry of Industry, Mine and Trade (MIMT)
5	Head	Government	Food Industry Department of Consumers and Producers Protection Organization, MIMT
6	Expert	Government	Food Security sub-Committee of the High Council of Health and Food Security, Ministry of Health and Medical Education (MOHME)
7	Respected Advisor	Academia	Infrastructure and Transformation in the Health System (MOHME)
8	Head	Government	Secretariat of Article 37 of the Fifth Development Plan (Office of Non-Communicable Diseases Management), MOHME
9	Head	Government	Community Nutrition Office, MOHME
10	Dean	Academia	National Nutrition and Food Technology Research Institute (NNFTRI)
11	General Director	Government	Office of Supervision of Value Added Tax (VAT) Administration, Tax Affairs Organization, Ministry of Economic Affairs and Finance
12	Head	Government	Fiscal Policy Research Office, Deputy Minister of Economy, Ministry of Economic Affairs and Finance
13	Director	Government	Department of Trade and Supportive Policies, Agricultural Planning, Economics and Rural Development Research Institute (APERDRI), Ministry of Agriculture-Jihad
14	Head	International org.	World Health Organization (WHO) regional office in Iran
15	President	Civil society	Iran’s Scientific Association for Healthy Food and Nutrition
16	Secretary	Industry	Federation of Iranian Food Associations
17	Secretary	Industry	Iranian Food Science and Technology Association
18	Head	Civil society	Iranian Nutrition Association
19	Faculty member	Academia	National Nutrition and Food Technology Research Institute

In Iran, the fiscal policies for SSBs are divided into two parts of the value-added tax (paid by the consumer) and taxes on production and import (paid by producers and importers of SSBs). The most important concepts that emerged from the interviews are categorized in the following three sections (Table 2):

**TABLE 2:** Themes and sub-themes extracted from the interviews regarding fiscal policies on carbonated soft drinks or SSBs in Iran.

Theme		Sub-theme
Fiscal policy development	Facilitators	Pattern of the disease (G, A)* The necessity of public access to healthy foods (G) The supply of energy required by people (CS)* Existing policies in the field of employment and economics and the GDP rate (G, A)
	Barriers	Economic sanctions (A) Scarcity of scientific documents for evidence-informed policymaking (G) Contradictions in the existing laws (I)* Involvement of different organizations and weak collaboration (G)
Fiscal policy implementation	Facilitators	Inclusion of policies on harmful goods in the laws (G) Creation of an opportunity for the Ministry of Health and Medical Education to list harmful products (G) Allocation of a part of the revenue to the treatment of diabetes and raising of awareness in the society (G)
	Barriers	Scattered laws (I, A) Absence of an authority (G) Poor supervision and inadequate enforcement guarantee (G) Lack of transparency in laws (G) Inadequate executive infrastructure (G) Controversial regulations (I) Low pricing by the Consumer and Producers Protection Organization (A) Inadequate participation of different stakeholders and absence of inter-organizational collaboration (A, G, and CS) Concerns of the Ministry of Industry, Mine and Trade about production stagnation (G) Industries’ reaction to the term “harmful” for their products (G) Limitation in the food production technologies (I) Prioritizing economic interests over health in the industrial sector (G) Failing to conduct a needs assessment for policymaking (A) Failing to review harms associated with harmful products (G, A) Overlooking of consumption habits in decision-making (G, A) Scarcity of prospective studies in the country (G) Failure to update tariffs (G) Poor supervision (A) Inadequate deterrence of the tax or duty (A)

*G, A, I, and CS are indicative of government, academia, industry, and civil society interviewees, respectively.

#### Facilitators of fiscal policy development and implementation

The first policy-making step in reducing the consumption of SSBs is to find a legal solution regarding the taxation of SSB import and production. This responsibility was carried out by the Office of Non-Communicable Diseases Management of MOHME. One of the governmental interviewees said:

*“For example, in the case of SSBs, there is a plan by the Ministry of Economy and Finance to take value-added taxes from SSB industries, and we approved and communicated it to the manufacturers” (G)*.

In addition to sales tax and VAT, duties are also levied on SSBs as specified in the Budget Law:

*“You can see that SSBs make up a dedicated income item in the Budget Law. In addition to these taxes, other taxes must be paid whose rate changes each year. This is not a VAT. It’s a specific type of tax or duty that is included in the Budget Law” (G)*.

The pattern of diseases throughout the country, the necessity of public access to healthy foods, and the supply of energy required by people were some factors mentioned by the interviewees, which affect the fiscal policies’ formulation. Other factors, including the current economy and employment policies in and the GDP rate play a role in the development of fiscal policies *(A, I)*.

The establishment of the Supreme Council of Health and its secretariat (responsible for addressing issues concerning the taxation of harmful goods), the inclusion of taxation policies on harmful goods in the related laws, creation of an opportunity for MOHME to list harmful products, allocation of a part of the revenue to the treatment of diabetics, and raising awareness in the society were some strengths of the fiscal policies mentioned by the interviewees. More collaboration between different organizations will help with evidence-based decision-making and facilitate the implementation of policies *(G)*.

#### Barriers of fiscal policy development and implementation

The scarcity of scientific documents for evidence-informed policymaking and contradictions in the existing laws (e.g., tax exemption for sugar) were some of the barriers mentioned by some interviewees for policy development. In Iran, different organizations are involved in the formulation of fiscal policies. Some of them are responsible for pricing such as the Consumers and Producers Protection Organization, Ministry of Economic Affairs and Finance, and Ministry of Industry, some support the Management Office of NCDs affiliated to MOHME, interfere in expert panel discussions, generate scientific evidence, and enforce mandatory laws, and some others like Ministry of Industry and Ministry of Agriculture Jihad examine the consistency of policies with needs. Sporadic policies and separate decisions by organizations in the past were among the barriers mentioned by interviewees for policymaking. But, at present, specific committees make decisions based on the consensus with more participation of the stakeholders. Economic sanctions have also been mentioned as one of the effective factors in the policymaking process *(G, A)*.

The interviewees believed that scattered laws and the absence of authority have led to reduced supervision, poor implementation, and inefficient regulations. There are currently two laws concerning SSBs; one in the Budget Law and the other in the Development Plan. It was proposed that these laws must be integrated, and taxes on unhealthy consumer products must be enacted with one Article and one Note. One interviewee said:

*“We said that these taxes and duties on harmful products must be integrated to a dedicated clause in the permanent VAT Act, instead of being addressed in the Budget Law, the VAT Act, and the Development Plan. If we merge these laws, we can have effective control and there will be a powerful authority” (G)*.

One of the challenges in the implementation of fiscal policies is the absence of authority. The Iranian National Tax Administration is in charge of tax affairs and tax collection. However, titles such as “duties” or “quasi-duties” do not have a dedicated authority. One interviewee mentioned:

*“One of the implementations concerns in the past was that some put SSBs in the Budget Law or a dedicated budget item, saying that the producers and importers must deduct this duty and pay it to the account of the dedicated budget item. Well? As duties, or other types of charges, tax or quasi-duties or whatever, these had to be paid to a dedicated account by the producers or importers. But there was no authority to supervise and examine their collection. These problems were resolved when the Iranian National Tax Administration undertook collecting these duties” (G)*.

Inadequate enforcement guarantees, lack of transparency in the laws, inadequate executive infrastructure, and poor supervision over harmful products in the industry were the other implementation challenges mentioned by the interviewees. On the other hand, there are some exceptions to taxation tariffs. The loopholes and exceptions pave the way for industries to bend, and ultimately, violate the laws. Conflicting regulations may lead to abuse and their use for personal interests. Many people also rely on tax evasion techniques. Meanwhile, some laws become void with the adoption of new laws. At the moment, the implementation of tax collection is short of the necessary commitment *(A, G)*.

Even though compilation of tax policies on unhealthy food products like SSBs is evidence-based and legal paths for its adoption is specified, there are still executive problems for implementation of these rules, as one of the experts said:

*“Taxation policy on unhealthy food products is evidence-based. In many countries of the world, it’s done, but it has limitations, e.g., to increase your price up to 10%; this price increase will be imposed on the consumers” (A)*.

Other implementation challenges mentioned by the participants were the low pricing by the Consumers and Producers Protection Organization, absence of inter-organizational collaboration, sporadic activities by the organizations, and the concerns of the Ministry of Industry about production stagnation. Flat-rate pricing of food products and the industry’s resistance to the term “harmful” for their products were other challenges in this area. In other words, the industrial sector does not allow for some of its products to be categorized as “harmful,” because they believe this will harm the interests of the sector. Therefore, they resist the implementation of such laws. The other point mentioned by the industry section was related to the existing production technologies:

*“Some factories lack the technology for improving product quality, which can create problems in the production line” (I)*.

Inadequate participation of different stakeholders was the other challenge mentioned by some of the participants *(A)*.

Inadequate participation of different stakeholders and prioritizing economic interests over health in the industrial sector, as well as the absence of MOHME and the private sector in the Market Regulatory Committee and the Targeted Subsidy Plan were identified as the main weaknesses of the fiscal policies in this area *(A, G)*.

Other weaknesses included failing to conduct a need assessment for policy-making, failing to review risks associated with harmful products, and overlooking the consumption habits in decision-making. Some of the reasons for the inefficiency of this law in the interviewees’ opinion were scarcity of prospective studies in this policy, failure to update tariffs, poor supervision, and inadequate deterrence of tax or duty *(A, CS)*.

### Experts’ viewpoints at the workshop

At the workshop, the attendees were informed of the data collected about the fiscal policies on SSBs and their strengths and weaknesses, SSB consumption, and CPI trend during the last years. Then each participant expressed his/her opinions and recommendations. One of them believed that the increase in milk prices was much greater than the increase in soft drink prices during the studied period. Elimination of milk subsidy and using subsidized sugar for SSB production resulted in high consumption of soda in many households, especially low-income ones. It has been recommended to earmark part of the SSB tax revenues for public health promotion *(G)*.

Another issue was that the custodian of tax was not explicitly specified in the law. For effective implementation, it has been recommended that before the formulation and development of fiscal policies, needs assessment studies should be conducted to determine the required policies. Another issue mentioned by one of the attendees was related to determining health threatening foods, which should be done according to scientific methods by an independent working group based on consensus. These items should be gradually introduced one by one in the law with precise and clear criteria by the responsible group. It has been stated that taxes on health threatening foods should be incorporated in the VAT Act as taxes on specific goods *(A, G, CS)*.

The other important issue mentioned by one of the academic participants was top-down approaches in formulation of fiscal policies. He believed that the authorities in-charge usually do not follow scientific and appropriate approaches. It is needed to engage, accompany, and collaborate with all stakeholders, especially those affected by the policy from formulation to implementation using the maximum capacities and data available. One of the special groups that should be considered is manufacturers. If they are involved in the policy making process, we can expect a good reaction and the least resistance in policy implementation *(A)*.

Furthermore, some of the participants stated that cross-sectoral cooperation with a continuous, national, inclusive, coherent and balanced approach and avoiding insular actions by relevant organizations are needed to decrease challenges regarding the implementation of these policies. The exact needs of the community should be targeted, too. In response to the industry concerns about factory bankruptcy and worker unemployment, one participant said that successful experiences across the world do not confirm this. To address this concern, taxes should be applied over time so that industries have enough time to adapt confirm and choose appropriate and alternative solutions. Industries can produce healthier and alternative products, use appropriate selling strategies, and find alternative markets such as exporting *(I)*.

All participants agreed that regular monitoring and evaluation plans should be considered in the policy making process. One of the participants suggested that sugar tax exemption must be eliminated and policy making must be accompanied by public education, prohibition of unhealthy food advertisement, and educational campaigns. One should keep in mind that by employing good advocacy strategies, delivery of clear messages and good compliance will be assured *(A)*.

## Discussion

This study explored Iran’s fiscal policies on SSBs and their weaknesses and strengths in addition to recommendations and future implications. It also described the household expenditure and the price index trends of SSBs. Although almost two decades have passed since the enactment of the taxation law on health threatening products in Iran, our results showed that the implementation of this law in the first years is not successful as expected, because evidence indicates that the household purchase of SSBs has not been changed considerably, meanwhile, the collection of tax revenue from SSBs has decreased drastically after almost 1 year of taxation policy enacted in 2013. Some challenges in terms of the policy formulation and implementation were responsible for its weak implementation as stated by the interviewees in this study. However, evidence suggests that fiscal measures such as taxes and subsidies can shift the consumers’ purchase habits and promote dietary change. Moreover, they are effective interventions to address NCDs (12, 30, 41). The most current product subject to the tax in the world and the only one in Iran is SSBs. Eight countries in EMR have introduced different amounts of taxes on SSBs [from 50% in the Persian Gulf Cooperation Council (GCC) to 20% in Iran] (28). One review by Hagenaars showed that among the unhealthy foods, the taxation of SSBs was the most appropriate and realistic from a policy making perspective, which was common across different countries except for Denmark (42).

The present research findings revealed that the proportion of household expenditures on SSBs has sharply increased from 2011 to 2013 and then remained almost unchanged. This proportion was slightly lower in rural households than in urban households. Due to the non-implementation of the law (40), it seems that the increase in the price of soft drinks and as a result, the increase in household expenses was mostly caused by inflation in the evaluated time period, and the deflated real price trend confirms this. In contrast, studies in South Africa showed that the prices of taxable SSBs have been increased and purchases of unhealthy SSBs and sugar intake consumption from SSBs have been decreased as a result of implementation of 10% tax, especially among the lower socioeconomic groups and the subpopulations with higher SSB consumption. Kantar Europanel data on monthly household purchases among a sample of South African households from all nine provinces were used to obtain per-capita sugar, calories, and volume from taxable and non-taxable beverages purchased before and after the HPL (Health Promotion Levy) announcement and implementation (43), whereas, we used the SSBs price and household expenditure data from ISC during the last decade. In Kazakhstan, one modeling study indicated that despite the increase in the price of SSBs over time, the proportion of household expenditures on these products has also increased (44). Among the different types of excise taxes, specific excise taxes which were used in Iran, are likely to be more effective than *ad valorem* excise taxes, because they increase the price of all taxed foods and beverages by the same (absolute) amount, and Therefore, consumers are motivated to substitute a product with a cheap tax (17, 38, 45).

Since there is no data regarding the households’ SSB consumption rate, we used the households’ expenditures on SSBs as a proxy of the consumption amounts. The price elasticity influences the extent to which a potential tax will be effective in reducing SSB consumption. A systematic review of studies in the U.S. revealed that a tax that raised 20% of the price of SSBs with the average price elasticity of demand of −1.21 would reduce the overall consumption by 24% (19). Our results are not consistent with the findings of other studies, which demonstrated that taxation and increasing the price of SSBs are effective tools for reducing their consumption (46, 47). In contrast, following the implementation of 50% Sin Taxes on soft drinks in 2017 in KSA, the soda prices increased and the annual purchases (in volume per capita) of soda and energy drinks reduced in 2018 compared to 2016 (48). The sales volume of SSBs decreased sharply with the implementation of Sin Taxes from 2010 to 2017 (30). The low amount of taxes on SSBs in Iran may not have the necessary deterrent to reduce their consumption. In the United States, the potential impact of a nationwide penny-per-ounce excise tax on SSBs showed a 15% potential reduction in SSB consumption among the adults aged 15–64 years (49). Low income households in the rural areas of Mexico had higher income elasticity and lower consumption due to increase in the price of SSBs (50). This was similar to lower income deciles in the rural areas of Iran where low-income households appeared to have slightly greater decline in SSB consumption.

The other factor that discourages purchasing a product is that people’s awareness of the tax on health threatening products (51). So, educating people regarding the cause of taxation besides increasing their knowledge on the risk of overconsumption of harmful foods and improving their food choice could increase the effectiveness of fiscal policies on decreasing the consumption of unhealthy food products. Besides pricing strategies, food labeling is another food policy that guides consumers to buy healthier food products and affects their purchasing behavior (52).

Iran’s Islamic Parliament Research Center in 2015 reported that the gradual change of people’s taste and food culture and increase in the consumption of fried and fast foods in Iran are among the reasons for their increased tendency to consume SSBs (53). The other reason for increasing the households’ expenditures on SSBs could be inappropriate implementation of the taxation policy. We found that the largest tax revenue belonged to the first year of taxation on SSBs (i.e., 2013); however, it decreased over time. Furthermore, the formulation and implementation of fiscal policies encountered some challenges; it seems that improper implementation of the tax policy on SSBs in Iran is responsible for inconsistency in the present research results. However, purposive and snowball sampling were used for sampling in the qualitative phase in which the representativeness of the sample is not guaranteed and the subjects that the researcher can obtain rely mainly on the previous subjects that were observed. Prior personal contacts, sample seed diversity, building trust in face-to-face interviews, and persistence (within reason) are helpful to delivering sample diversity in snowball samples (34).

Review of the literature revealed that public health and economic motivations co-exist in the soda tax policy process (26, 54, 55). Health issues and pattern of the diseases across the country were the most cited issues affected the SSB fiscal policy development in Iran. It was similar to Mexico and Hungary, where high rates of chronic diseases and urgent need to prevent unhealthy eating were found to be more critical than financial reforms (13, 54, 56), while in other countries such as Saudi Arabia, financial reforms were the main issue that helped the pass of tax policy (26, 55).

In this study, job loss was a concern mentioned by the industrial sector as an implementation challenge of fiscal policies. In Australia, the beverage and sugar industry highlighted pressure for SSB tax and successfully lobbied to keep the SSB tax off the table (57). Another study demonstrated that soda taxes interfere with the interests of the food and soda industries and exert strong lobby efforts for policies in favor of their interests (58). However, a systematic review showed no significant job loss and no robust evidence for the negative macroeconomic impacts of fiscal policies on SSBs (59). It also highlighted that revenue must be used for complementary initiatives such as employment generation or livelihood training for those affected (59). Limitations in the existing production technologies and reformulation of healthy food products were other challenges mentioned in our study, while food companies in the United Kingdom reformulated their products in anticipation of a SSB tax that would enter into force 2 years after the announcement (60). It has been recommended that tax on SSBs encourages manufacturers to reformulate their products and produce more healthy foods.

Absence of authority and lack of transparency were considerable barriers to SSB tax adoption and implementation in Iran. However, it has been demonstrated that framing realistic and well-articulated public health and budgetary objectives are considered as a matter of transparency and credibility of such laws, which are deemed necessary for soda tax adoption (61). The other challenging issues were contradictory regulations and scattered laws. Subsidy on sugar, like the other countries in EMR (52), was another challenging issue that has affected the pricing of SSBs in Iran. WHO recommends eliminating subsidies for sugar as well as fats and oils (28). Weak collaboration between different sectors was another challenge mentioned in the present study. Successful experience of public health product tax in Hungary is a good example of the shared concerns and collaboration between different sectors in health and finance and their respective services (13). Roache and Gostin indicated that advocacy among local organizations, lobbyists, politicians, and celebrities has played a key role in adopting many existing soda taxes in different countries (62). The advocacy and communication campaigns applied in KSA were responsible for a slight reduction of SSBs up to 2016 though a gradual reduction was seen after introduction of Sin Taxes (30).

Despite the international evidence, the lack of needs assessment studies for policymaking in the current context is one of the weaknesses of fiscal policies on SSBs in Iran. Although different studies have demonstrated the effect of SSB taxation on reducing their production, the existing context of each country should be considered when designing such policies. The systematic reviews have shown the positive effects of tax policies on reducing the purchase and consumption of taxed beverages and prevention of NCDs (17, 18, 63–66). Saudi Arabia has an evidence-based rationale for SSB tax structure to ensure sustainability and frustrate industry opposition (26). Evidence indicates that unlike Iran (15% tax rate), high tax rates (50%) have led to a decrease in the annual growth rate of soft drink sales volumes in Saudi Arabia and its neighboring countries, whereas political powers in France, particularly in the economic sector focus more on SSB taxation, instead (67), while in the United States, civil society may be more influential (68). Giving the time for the industry to adapt to these reforms is extremely complex, especially since the evidence obtained around the world does not indicate such a necessity.

## Conclusion

Effective prevention of NCDs and other diseases related to excess consumption of SSBs depends on adopting proper policies. The present research findings proposed some suggestions for increasing the effectiveness of financial policies in preventing and reducing NCDs in Iran, including the need for scientific consultation with scholars and academics, outsourcing the provision of evidence needed to formulate some policies, resolving controversial regulations, structuring the path of financial policies from development to implementation and evaluation, strengthening the monitoring and evaluation of policies, research on the formulation of healthy and functional beverages, the price elasticity of specific products and tax cost-effectiveness, cross-sectoral cooperation through a continuous, national, inclusive, coherent and balanced approach, attracting all stakeholders, providing the necessary infrastructure for production, policymaking together with public education, prohibition of advertising health-threatening goods, educational campaigns, and planning to change the policies of consumerism.

Other important issues, which could be considered in the policy, include allocation of portions of the collected tax as a milk subsidy to low-income families, increasing the export of soft drinks, allocation of subsidies to the employment sector and the industries that produce healthy foods in, and supporting innovative technologies and science-based companies in the beverage industry, which will lead to the production of functional drinks. Removing subsidies on sugar, at least for health threatening products such as SSBs, has been emphasized by the experts in our study together with taxes for eliminating the existing contradictory laws. In countries with the same context to Iran, comprehensiveness of the law, establishment of the minimum effective tax rate, integration into value added tax or other similar systems, and continuous evaluation and monitoring of law enforcement are suggested, especially when the industry has a lot of power.

### Limitations

The current study is the first one that evaluate the implementation of existing fiscal policies on SSBs in Iran and indicates its strengths and weaknesses from the perspective of the key informants. The present study did not intend to analyze the SSBs tax policy process and contextual determinants. Although these are important in policy analysis, they are out of the scope of this study. Moreover, some laws and fiscal policies were unavailable. The existence of numerous stakeholders in different ministries and having contradictory perspectives make reaching a consensus some difficult. The other limitation was related to the difficulty of coordinating with some stakeholders to conduct an interview. Moreover, the retrospective nature of the study and the use of existing data did not allow evaluating the impact of tax policy on SSBs purchase and consumption of the community. Using the expenditure data to estimate the consumption of SSBs in the studied population is a limitation that makes interpretation of the data cautious. Because as the price increases, the costs also increase which may not be related to the amount of SSB consumed.

## Author contributions

FM-N and AZ conceived and designed the study, participated in the literature review, data extraction, and interpretation of the results. DG wrote the first draft of the manuscript and involved in secondary data gathering. AH-R, MRKh, and SFA-P contributed to the qualitative data gathering. DG and AH-R contributed to the qualitative data analysis. FE and MA involved in extracting data from the reviews. MB conducted the analysis of secondary data. All authors read, commented on, and agreed to the final version of the manuscript.

## Acknowledgments

This project was implemented in the National Nutrition and Food Technology Research Institute (NNFTRI) with the financial support of the World Health Organization’s (WHO) Regional Office and the cooperation of the Community Nutrition Office of MOHME under the supervision of Nasrin Omidvar from the Community Nutrition Department of the Faculty of Nutrition Sciences and Food Technology. The authors would like to express their gratitude and appreciation to the esteemed representative of the WHO in Iran, Samin Sedighi and the esteemed colleagues of the WHO Office in Iran, especially Mansour Ranjbar, as well as Sonia Tahmasebi, and Marzeyeh Soleymani Nejad. We also appreciate Ebrahim Parvin for editing and proof-reading the final manuscript. Finally, the authors thank the esteemed representatives of the following governmental and non-governmental organizations, which contributed to conducting this research with their valuable comments:

■ General Directory for Food and Beverage Products, Iranian Food and Drug Administration (IFDA).
■ Honorable Advisor to the Minister of Health on Infrastructure and Health System Transformation.
■ Secretariat of the Supreme Council of Health and Food Security, MOHME.
■ Secretariat of Article 37 of the Fifth Development Plan (Office of Non-Communicable Diseases Management), MOHME.
■ General Directory of Supervision of Value Added Tax (VAT), Tax Affairs Organization, Ministry of Economic Affairs and Finance.
■ General Directory of Non-metal Industries of the Ministry of Industry, Mines and Trade (MIMT).
■ Food Industry Department of Consumers and Producers Protection Organization, MIMT.
■ General Office of Research and Financial Policies, the Ministry of Economic Affairs and Finance.
■ Department of Trade and Supportive Policies, Agricultural Planning, Economics and Rural Development Research Institute (APERDRI), Ministry of Agriculture-Jihad.
■ Federation of Iranian Food and Nutrition Associations.

## Appendix

Appendix 1 | Interview protocol for phase 3: open questions.

- What are the current financial policies in our country that are related to nutrition?
- What are the financial policies that specifically affect the prevention of non-communicable diseases?
- Are there any laws, bills and regulations in the country that can pursue the goal of reduction of non-communicable diseases?
- If the answer is yes, could you please explain them to us?
- Can you please explain more specifically about the tax policies related to nutrition in the country?
- Can you please explain your opinion about the subsidy allocation policy in the country?
- In your opinion, what are the strengths of these policies in general?
- In your opinion, is there a specific policy that has more strengths in reducing non-communicable diseases?
- Do you think there are any weaknesses for this type of policy?
- If the answer is yes, what weaknesses?
- Are these policies implemented correctly?
- In your opinion, is the information of the policymakers sufficient and based on evidence to formulate such policies?
- In your opinion, what are the strengths and weaknesses of the implementation of these policies?
- What is your suggestion to improve the situation of developing such policies?
